# Heat Stress as a Barrier to Successful Reproduction and Potential Alleviation Strategies in Cattle

**DOI:** 10.3390/ani13142359

**Published:** 2023-07-19

**Authors:** Imran Khan, Ayman Mesalam, Yun Seok Heo, Seo-Hyun Lee, Ghulam Nabi, Il-Keun Kong

**Affiliations:** 1 Department of Biomedical Engineering, College of Engineering, Keimyung University, 1095 Dalgubeol-daero, Dalseo-gu, Daegu 42601, Republic of Korea; imrangnu@gmail.com; 2 Department of Theriogenology, Faculty of Veterinary Medicine, Zagazig University, Zagazig 44519, Egypt; aymanmesalam@gmail.com; 3 Department of Premedicine, School of Medicine, Keimyung University, 1095 Dalgubeol-daero, Dalseo-gu, Daegu 42601, Republic of Korea; 4 Department of Animal Science, Division of Applied Life Science (BK21 Four), Gyeongsang National University, Jinju 52828, Republic of Korea; 5 Institute of Nature Conservation, Polish Academy of Sciences, 31-120 Krakow, Poland; ghulam@iop.krakow.pl; 6 The King Kong Corp., Ltd., Gyeongsang National University, Jinju 52828, Republic of Korea

**Keywords:** heat stress, reproduction, pregnancy, embryo development

## Abstract

**Simple Summary:**

The impact of heat stress on reproduction is complex and multifactorial and is compounded by growing challenges due to climate change. Both animal welfare and fertility are vulnerable parameters easily affected by heat stress. Heat stress leads to a marked decrease in the developmental competence of oocytes and the fertilizing capacity of spermatozoa, leading to a declining reproduction rate and losses for the cattle industry. There is an urgent need to find viable methods of alleviating the effects of global warming on animal welfare and fertility and, accordingly, guard the reproductivity, productivity, and profitability of cattle farms.

**Abstract:**

In recent decades, the adverse effects of global warming on all living beings have been unanimously recognized across the world. A high environmental temperature that increases the respiration and rectal temperature of cattle is called heat stress (HS), and it can affect both male and female reproductive functions. For successful reproduction and fertilization, mature and healthy oocytes are crucial; however, HS reduces the developmental competence of oocytes, which compromises reproduction. HS disturbs the hormonal balance that plays a crucial role in successful reproduction, particularly in reducing the luteinizing hormone and progesterone levels, which leads to severe problems such as poor follicle development with a poor-quality oocyte and problems related to maturity, silent estrus, abnormal or weak embryo development, and pregnancy loss, resulting in a declining reproduction rate and losses for the cattle industry. Lactating cattle are particularly susceptible to HS and, hence, their reproduction rate is substantially reduced. Additionally, bulls are also affected by HS; during summer, semen quality and sperm motility decline, leading to compromised reproduction. In summer, the conception rate is reduced by 20–30% worldwide. Although various techniques, such as the provision of water sprinklers, shade, and air conditioning, are used during summer, these methods are insufficient to recover the normal reproduction rate and, therefore, special attention is needed to improve reproductive efficiency and minimize the detrimental effect of HS on cattle during summer. The application of advanced reproductive technologies such as the production of embryos in vitro, cryopreservation during the hot season, embryo transfer, and timed artificial insemination may minimize the detrimental effects of HS on livestock reproduction and recover the losses in the cattle industry.

## 1. Introduction

Cattle are homeothermic animals that maintain a specific constant body temperature; however, due to variations in environmental temperatures and humidity, their body temperature changes [1]. Cattle, in general, and some classes like European breeds in particular, are highly threatened by global warming [2,3]. Heat tolerance has a close relationship with cattle breed [4]. The most sensitive and vulnerable breed to HS is the Holstein Friesian breed [5]. Moreover, British breeds have a higher vaginal temperature, an indicator of increased environmental temperature, compared with Bonsmara crosses at the same temperature and humidity index [4]. Depending on their response to HS, cattle breeds are marked as high or low immune responders, responding more or less severely to HS, respectively. Due to HS, there is a clear methylation pattern of the promotor regions of blood mononuclear cells (BMCs) between high-immune-responder and low-immune-responder cattle. High-immune-responder cattle have a greater response to HS compared with low-immune-responder cattle, which curtails the detrimental effect of HS [6].

Similarly, the two breeds of bulls called Angus and Nellore have different patterns of blood methylation due to HS. The methylation pattern in the Nellore breed is more focused on defensive and survival pathways in regard to HS, while the Angus breed’s methylation pattern is less focused on survival [7]. HS has transgenerational effects on cows. These transgenerational effects include maturation, stillbirth, and the yield and concentration of fat. From all this information, it is evident that HS during pregnancy affects at least three generations, regardless of their life circumstances [8]. Likewise, embryo and fetal development are affected during pregnancy in the hot season, and this leads to changes in the intrauterine growth, metabolism, epigenomes, thermotolerance, and immune response of the resulting offspring. Due to all these changes in genome and phenotype, the developing offspring faces lower milk production and survival rates in the next generation [9].

Heat stress (HS) occurs due to elevated ambient temperatures above the thermoneutral zone, along with high humidity and slow air movement [10,11]. The most common index used to evaluate the degree of HS is the temperature–humidity index (THI), which can be used to predict the milk yield of dairy cows [12]. HS, particularly during summer, disrupts numerous reproductive processes in dairy cows worldwide. A temperature in the range of 5 to 25 °C is considered the best temperature for cattle’s overall performance [13,14]. However, in summer, the upper limit of this temperature range is crossed in tropical and subtropical regions, resulting in the disruption of the reproductive performance of cattle, both in summer and in the following season due to the delayed effect of HS [15,16]. In Florida, a rectal temperature of 39 °C due to a 29.7 °C environmental temperature causes mild hyperthermia, while a temperature above 31.4 °C causes true hyperthermia [12]. High rectal temperature is considered to be a major cause of low reproduction [17]. In the US, the rates of reproduction and milk production in cattle sharply decline in the hot season due to HS, leading to losses of approximately USD 900 million in the dairy industry every year [18]. In addition to the metabolic stress associated with high milk production in dairy cows, seasonal effects on fertility have been noted in a large number of reports [19].

High temperatures and humidity are the main factors responsible for HS, which, in turn, adversely affect reproductive performance [16]. Respiration greater than 60/min and a rectal temperature higher than 39 °C cause heat strain [20], which affects the fertility and milk production of cattle [18]. Cattle’s survival and productivity in hot climates and hilly areas depend on their grazing; in areas where they are exposed to a hot and humid environment, they experience an increase in body temperature from the thermoneutral level and, hence, HS. Unfortunately, approximately 60% of dairy farms worldwide are located in heat-stress environments [21]. The effect of THI differs across countries. Evaluating the consequences of HS on milk production across three European regions, Slovenia showed a lesser effect of temperature and humidity on milk production compared to Spain, Belgium, and Luxembourg [22]. HS severely affects the fertility of dairy animals. In one study, the conception rate in the first service decreased from 25 to 7% when the temperature increased from 29.7 °C in April to 33.9 °C in July [23]. It was found that from 2010 to 2015, the average temperature in summer increased by 1.5 °C, leading to a further 5% decrease in the conception rate [24]. This increased global warming greatly affects the normal body temperature of cows, and it was found that metabolic heat production is increased between 2- and 4-fold due to global warming, which negatively impacts the reproduction of cattle [25]. The developmental competence of oocytes is reduced by HS, which, in turn, reduces fertility, and this is one mechanism through which the reproduction rate of animals is reduced during the hot summer season [26]. Different mechanisms like radiation, conduction, and convection are used by mammals to respond to HS; however, when this thermoregulatory system fails, evaporative methods like panting and sweating are used to combat HS and maintain a normal body temperature [25]. However, at high environmental temperatures and in humid conditions, these body temperature maintenance mechanisms are not sufficient and hence ultimately lead to HS [10,27].

The objective of this review is to shed light on the mechanisms of heat stress in regard to the fertility of cattle. We also attempt to cover the available strategies employed to relieve the effects of HS on reproduction in the changing climate scenario.

## 2. The Impact of Heat Stress on Cattle Production

Cattle production losses from HS were estimated to represent 98% of the value of the production of meat and milk in 2005 [28]. In dairy cows, milk production is related to the acceleration of the metabolic rate, resulting in more metabolic heat. The metabolic rate of a cow producing 31 kg of milk is approximately 17% higher than that of a cow producing 18 kg of milk per day [29]. The impact of HS on milk production per tropical livestock unit (an adult animal weighing 250 kg) for the top global milk producers is predicted to diminish by 25% by the end of the current century compared with 2005 [28]. Additionally, in the context of high greenhouse gas emissions, the adverse effect of HS on beef production per tropical livestock unit for the top ten beef producers is projected to decline over the same period [28].

The sympathetic–adrenomedullary system (SAM) and the hypothalamic–pituitary–adrenocortical axis (HPA) constitute the sympathetic autonomic and adrenal medulla, and this whole system is activated by HS. The hypothalamus is the regulatory point that responds to HS in cows. When the SAM system is activated, there is a higher secretion of catecholamines, adrenaline (A), and noradrenaline (NA) in the bloodstream, which, in turn, affects the functions of the internal organs. This system is activated on exposure to HS to cope with its detrimental effects [30]. The high temperature causes the high secretion of cortisol in the bloodstream of dairy cattle [31]. The immune system of cattle is affected by the prolonged secretion of cortisol, which results in the secretion of interleukin 6 (IL6) [32]. As a result of this situation, the immune systems of cattle are compromised and various diseases develop [33].

## 3. The Impact of Heat Stress on the Endocrine Factors Controlling Reproduction

HS affects fertility and inhibits livestock reproductive performance through hormonal imbalance. The main critical factors that control ovarian activity are the hypothalamic gonadotropin-releasing hormone (GnRH) and the anterior pituitary gonadotropins (FSH and LH). The effect of HS on gonadotropins is controversial [24]. HS suppresses the LH pulse amplitude and frequency and compromises its function in cattle, thus compromising the maturation of dominant follicles and decreasing estradiol secretion [34]. Moreover, a lower LH surge and lower LH tonic levels with HS impair ovulation and functional CL formation, leading to low progesterone levels [24]. In contrast, HS increases FSH secretion due to a marked decrease in plasma inhibin, leading to the growth of a larger number of follicles in the ovaries [35,36] that might cause double ovulation and, subsequently, twin calving during the summer season [24]. Furthermore, HS stimulates the pituitary gland to secrete the adrenocorticotropic hormone (ATCH), which stimulates the release of glucocorticoids that inhibit oocyte meiotic maturation [37].

## 4. The Impact of Heat Stress on the Estrous Cycle

The estrous cycle refers to the cyclic pattern of ovarian activity that enables female animals to establish pregnancy following mating, while the term estrus refers to the phase when a female is sexually receptive to males. A significant reduction in the fertility of cattle was reported during summer due to the effect of HS in increasing body temperature [24]. Moreover, follicle quality and hormonal balance are also changed under HS conditions, leading to declining estrus [38]. Similarly, the granulosa cells responsible for the production of estradiol are negatively affected by HS, which reduces the production of estradiol, resulting in the silencing of the estrous cycle in cattle [39]. The effects of HS on corticoids, the luteinizing hormone (LH), and plasma progesterone have been documented by various researchers [40,41]. Additionally, HS affects the hypothalamus and pituitary gland in cattle and changes the secretion of reproductive hormones [42]. Alterations in the concentrations of these hormones negatively affect the estrous cycle, which leads to reduced fertility among domestic animals in hot regions of the world [43].

Lactating cows are very sensitive to low fertility because lactation is associated with increased metabolic heat, which reduces the timing and strength of estrus, reduces the conception rate, and increases embryo mortality [44]. The estrus detection rate in dairy cattle is lower in summer than in winter and spring. Direct solar radiation and humidity, together with ambient temperatures, intensify HS in cattle and buffalo. Bharat Merino ewes exposed to HS have a shorter estrus duration due to the low production of estradiol as a result of HS [45].

## 5. The Impact of Heat Stress on Oocyte Competence

The quality of oocytes is reduced during summer due to HS, causing great losses for dairy producers [46]. The oocyte maturation period is short compared to the total life span of cattle; however, the exposure of oocytes to HS during this period causes fertility problems because HS disturbs the physiology of oocytes, leading to compromised reproduction [47]. It is known that HS negatively affects the hypothalamus of the pituitary gland. HS reduces the level of the luteinizing hormone (LH), leading to poor follicles and low-quality oocytes [39]. Similarly, follicles and oocytes exposed to HS are highly sensitive [48]. During summer, HS compromises the mRNA expression levels of various genes (*MOS*, *GDF9*, and *POUF51*) involved in the development of oocytes and their competence in various cell stages [49]. Poor-quality oocytes also affect the genes involved in transcription and embryonic development [49], leading to apoptosis and reduced embryo quality [50]. Bovine oocyte cytoplasmic and nuclear maturation are affected by HS, and oocytes collected in summer are of a lower quality than those collected in winter [51]. Similarly, oocytes collected in summer are more susceptible to heat stress than those collected in winter, leading to compromised reproduction [52]. During oocyte maturation, spindle fibers and microtubules are affected by HS, and the size of the meiotic spindle is reduced due to the deleterious effects of heat shock, which reduces oocyte maturation [53].

HS also alters the hormonal balance of cattle, which reduces oocyte quality [54]. In warm countries, the low fertility rate observed even in autumn is due to the delay in the effect of HS on steroid production, leading to low-quality follicles and oocytes. In the same way, during hot summers, progesterone levels and LH are adversely affected by HS in females, leading to poor follicles and low-quality oocytes [26]. It was found that the fertilization rate of normal cows declines from 83% to 37% due to the deleterious effect of HS on oocyte maturity [55]. Moreover, the conception rate is reduced by 20–30% in summer because of HS [56]. Poor oocyte quality due to HS results in a poor-quality embryo, leading to a low birth rate and compromised reproduction [34]. It has also been proven by various scientists that HS also reduces the developmental competence of blastocysts in vitro [57,58,59] (Table 1).

The reproductive capacity of cows declines due to the detrimental effect of HS on the process of oogenesis. When cattle are exposed to HS, abnormal hormones are released from the hypothalamus, leading to poor follicles and low-quality oocytes. These low-quality oocytes compromise fertility and reproduction [63].

## 6. The Impact of Heat Stress on Bull Fertility

Males are at a higher risk of the adverse effects of high environmental temperatures and relative humidity, which can reduce sperm production and decrease male fertility rates [64]. The process of spermatogenesis is very sensitive to HS. To maintain a specific physiological temperature below body temperature, most mammals, including cattle, have a special sac called the scrotum [65]. In order to protect the vulnerable developing sperm from higher environmental temperatures, the scrotum and testes have their own thermo-regulatory mechanisms [66]. The exposure of bulls to HS has an adverse effect on fertility through a reduction in antioxidants that can protect the developing sperm from oxidative stress or the adverse effect of reactive oxygen species (ROS) on sperm DNA [66]. Dairy bull semen quality and cattle fertility are negatively affected by seasonal HS and climate conditions [67]. Moreover, elevated temperatures cause mitochondrial dysfunction, hampering glucose oxidative metabolism and, subsequently, leading to the accumulation of ROS and increased lipid peroxidation that result in increased sperm primary abnormalities [68].

## 7. The Impact of Heat Stress on Semen Quality

Properties like morphology, motility, plasma membrane integrity, metabolic activity, and the capacity for acrosome reaction define the quality of semen [69]. In bulls, spermatogenesis is reduced by heat shock [70]. The number of live sperm cells is reduced upon the local heating of the scrotum in humans, and constant scrotal heat stress can impair semen quality [71]. HS mainly affects serum testosterone and LH levels, which were found to be decreased in heat-stress-exposed male guinea pigs [72]. Sperm counts and the number of motile cells are lower in summer than in winter and spring [68,73]. The exposure of bull semen to high temperatures causes aging and a reduction in quality and, hence, reduces the semen’s fertilization efficiency. Similarly, HS also reduces the motility and yield of sperm, and it takes 8 weeks for bulls to recover from such stress [74]. The quality of semen significantly affects fertilization, cleavage, and blastocyst development rates as well as the quality of embryos [75]. Similarly, the efficiency of in vitro fertilization is also affected by bull semen quality due to climate changes in tropical regions [76]. Further studies are required to verify this decline in the efficiency of semen due to HS, the molecular pathway involved, and methods for mitigating the adverse effect of HS on semen quality and reproduction.

## 8. The Impact of Heat Stress on Fertilization

The ability of spermatozoa to penetrate an oocyte and activate it to develop an embryo is called fertilization [77]. Successful fertilization consists of many steps, such as the penetration of the cumulus oophorus by the sperm, the interaction of the sperm with the zona pellucida, sperm–oocyte fusion, oocyte activation, the de-condensation of the sperm nucleus, and pronuclear formation [78]. HS has an adverse effect on oocyte fertilization as higher temperatures cause oocytes to degenerate, reducing fertilization [79]. When ewes were exposed to heat shock 12 days before breeding, their fertilization rate was reduced [80]. Exposure to HS during fertilization can be lethal to sperm, causing oxidative stress and blocking polyspermy, which is likely a major factor contributing to the reduced developmental competency of fertilized zygotes at elevated temperatures [81]. In cattle, if sperm are exposed to HS of approximately 40 °C for 30 min, this will cause a significant decline in their viability, integrity, and fertilization efficiency [82].

HS not only affects fertilization and the reproduction rate in vivo but also reduces embryo production in vitro by reducing the quality of oocytes obtained from ovaries recovered from slaughterhouses, resulting in a sharp decline in the in vitro production of cattle embryos [83]. Fertilization also depends on the duration of exposure of oocytes to HS, and oocytes exposed to HS for 12–24 h during their maturation period exhibit reduced fertilization and blastocyst development rates [84]. Furthermore, HS of 40.5 °C reduces oocyte maturation, leads to low fertilization, and compromises bovine embryo development in vitro [61]. Although HS reduces the rate of fertilization in cattle, it is not clear whether this decline in fertilization is mainly due to the oocyte quality or the semen quality. HS indirectly affects fertilization because heat shock causes the early maturation of oocytes [85] and, when an HS-exposed oocyte is fertilized at 24 h of maturation, sperm fertilize aged oocytes; hence, the rates of fertilization and blastocyst development are reduced. Heat stress induces the overproduction and accumulation of reactive oxygen species (ROS), leading to a loss of mitochondrial membrane potential, electron leakage, and the impairment of lipid peroxidation and sperm motility; lowering energy production; promoting premature capacitation; and reducing the sperm lifespan as well as fertilization and embryo development rates [70].

## 9. The Impact of Heat Stress on Developing Embryos and Newborns

In cattle, the activation of the embryo genome takes place after the first stage of cleavage (after the four-cell stage); hence, in this stage, the embryo is highly susceptible to any kind of stress, including HS [3]. The genome activation of bovine embryos and resistance to stress develop in the same cell stage (8–16 cells) [58]. Tolerance to HS is accomplished through the production of heat shock proteins (HSP) [59]. Although embryos may exhibit a certain degree of malleability to minor changes in temperature, sustained exposure to higher temperatures can reduce fertilization, implantation, and successful pregnancy rates [86]. The developmental efficiency of oocytes collected during summer with respect to the preimplantation embryo is lower than that of oocytes aspirated during winter [49]. Also, HS negatively affects the quality and survival rate of embryo development in vitro [62]. HS can affect embryos at any stage; however, early-stage embryos are more susceptible to HS [87]. Preimplantation embryos are particularly susceptible to, and can easily be disrupted by, HS [16]. This period is highly critical for the attachment of the developing embryo to the endometrium and, at this point, HS adversely affects the attachment of the embryo to the maternal tissues [88,89]. Before day 17 after insemination, the period during which interferon-tau (IFNT) is produced, embryos are more susceptible to HS, leading to embryo mortality and pregnancy loss because IFNT has the main role of sustaining a healthy pregnancy [90].

HS increases the concentration of ROS, which leads to damage to the developing embryo [91]. It was reported that the concentrations of ROS such as hydrogen peroxide (H_2_O_2_) and superoxide anion (O_2_^−^) were increased in vitro [92], which reduced embryo quality through apoptosis [93,94]. HS increases energy metabolism, mitochondrial activity, and oxygen demand in various tissues in the body, resulting in increased ROS production [95]. In hot seasons, HS not only affects the quality of embryos but also reduces their number, possibly due to its negative effect on oocyte quality and the number of recovered oocytes following superovulation, fertilization rates, and embryo quality [96]. In Louisiana and Wisconsin, embryo production is significantly reduced in summer because of heat shock [62]. In addition, HS adversely affects epigenetic factors and reduces the response of embryos to stress, resulting in poor-quality embryos [97].

A decreased gestation length has been reported following exposure to HS during gestation [29]. The exposure of dairy cows to HS during late gestation affects postnatal immune status and the performance of offspring and increases intestinal apoptosis, which decreases IgG absorption and impairs passive immune competence [98]. Similarly, retarded fetal growth was observed in lambs born from sheep exposed to HS during late gestation due to suppressed placental development, with a reduced cotyledonary mass and decreased mid-uterine artery blood flow [99].

## 10. The Impact of Heat Stress on Pregnancy Loss

In cattle, the embryonic period starts with the fertilization of the ovum by the sperm and lasts until differentiation (42 days), while fetal development ranges from day 42 to the birth of the fetus. Pregnancy loss is usually observed on day 35, before the fetal development stage [100], and the chance of miscarriage is very high with a twin pregnancy [101]. From the literature review, it is clear that HS is lethal to cattle reproduction and disturbs hormone levels. Decreased hormone levels not only negatively impact the estrous cycle in cattle but also cause miscarriage after pregnancy. Progesterone is the key hormone for the maintenance and development of the embryo and fetus for at least 6 months [102]. The relationship between progesterone and miscarriage was previously elucidated [103], and several studies reported that the progesterone level is very important for maintaining pregnancy. Similarly, reduced embryo survival and increased pregnancy loss are also associated with a declined concentration of progesterone in the blood circulation due to altered luteal function affected by HS [42]. Similarly, progesterone levels are reduced in buffalo upon HS, which affects the estrous cycle and compromises reproductive performance, especially in hot seasons [104]. Other possible reasons behind higher pregnancy losses during summer are the direct effect of HS on embryo survival and ovarian function. The pregnancy rate of cattle is reduced because ovarian function is unfavorably affected by heat shock [105]. The reduced pregnancy rate of cattle is related to early embryonic death caused by HS [106]. Additionally, specified heat shock proteins have been correlated with several pregnancy complications, including pre-eclampsia and fetal growth restriction [107]. Cows and sheep exposed to chronic and extreme heat have a reduced uterine blood flow that can result in the restriction of fetal growth and low placental and birth weights [108].

## 11. The Alleviation of Reproductive Compromise under Heat Stress

Lower reproductive efficiency under heat stress can be minimized by adopting appropriate scientific strategies involving physical amendments to the environment and the genetic improvement of heat-stress-tolerant breeds [109,110]. Moreover, it is possible to secure reproductive success during summer through the application of advanced reproductive technologies, including hormonal treatments, ovulation synchronization followed by fixed-time artificial insemination, and the use of embryo transfer, to improve the chance of pregnancy [111,112].

The effects of HS on the estrous cycle and the reproductive efficiency of cattle can be reduced by providing shade and air conditioning during the hot summer season to minimize losses to the cattle industry [113]. Three main methods are utilized to reduce the impact of HS, including low-volume, high-speed fans; high-volume, low-speed fans; and tunnel ventilation [114]. The efficiency of these cooling methods is measured in terms of milk production and conception rates, which show that milk production recovers in summer, but the conception rate is different, being 22% lower in summer, demonstrating that HS is lethal and cannot be restored through ordinary methods, thus requiring special attention [115]. The reason for this may be that cooling using ventilation without water spraying is not efficient for preventing hyperthermia in cows. This encouraged scientists to develop a cooling approach based on the spraying of water onto the skin of cows, followed by its evaporation via air from fans to effectively prevent hyperthermia [116].

Recent studies indicated that in summer, HS can alter gonadotropin, depress LH secretion, and decrease cow fertility [117]. A potential approach to ameliorate the adverse effect of HS is to administer a gonadotropin-releasing hormone (GnRH) at the onset of estrus in order to stimulate the release of a normal LH surge. A previous study reported that the treatment of heat-stressed cows with GnRH at the onset of estrus increased conception rates [118]. In addition, the deleterious effect of HS in lowering plasma progesterone could be minimized through the application of exogenous progesterone for 14 days post-insemination in the summer. It has been reported that exogenous progesterone supplementation with controlled intravaginal drug release (CIDR) on day 5 post-insemination for 13 days improved the summer fertility of cows and increased the conception rate by 6% [119].

The exposure of animals to HS during summer reduces the intensity and length of estrus and consequently increases the incidences of silent ovulation and anestrous females. Therefore, the use of ovulation synchronization and timed AI protocols are practiced to improve fertility and overall pregnancy rates in summer because all cows are inseminated without estrus detection [120]. Furthermore, the application of a synchronization program during the heat stress period increased progesterone levels and improved fertility [14,121]. Since the early stages of preimplantation embryo development involve high vulnerability to HS, the application of embryo transfer after bypassing the thermosensitive stages can be used at day 8 to ameliorate the undesirable effects of HS on cattle embryonic development [122].

Given that the exposure of oocytes to HS hastens germinal vesicle breakdown and leads to rushed oocyte aging [85], fertilization rates and embryo development can be improved by reducing the maturation time of HS-exposed oocytes from 24 to 19 h [123]. In an in vitro embryo culture, HS was responsible for a sharp increase in the level of ROS, leading to declined blastocyst development [39,124,125]. To study and counterbalance the effects of HS and ROS generated in culture systems in vitro, various antioxidants (ROS scavengers) are used [126,127,128,129,130]. In our previous research, we also used an antioxidant, coagulation-A, to counterbalance the deleterious effect of HS. We found that oocytes exposed to HS at 40.5 °C during in vitro maturation and treated with coagulation-A had higher levels of heat shock protein 70 (HSP70) and phosphatidylinositol-3-kinase (PI3K), which counterbalanced the ROS and shock produced as a result of HS and improved bovine blastocyst development in vitro [61]. Although various antioxidants can be added to a culture medium in vitro to counterbalance the deleterious effects of HS and produce good-quality embryos, which can then be preserved and transferred to the recipients, more work is required to identify an exact solution to this problem, especially in tropical regions where temperatures are high and summers are long.

## 12. Conclusions

**Figure 1:**
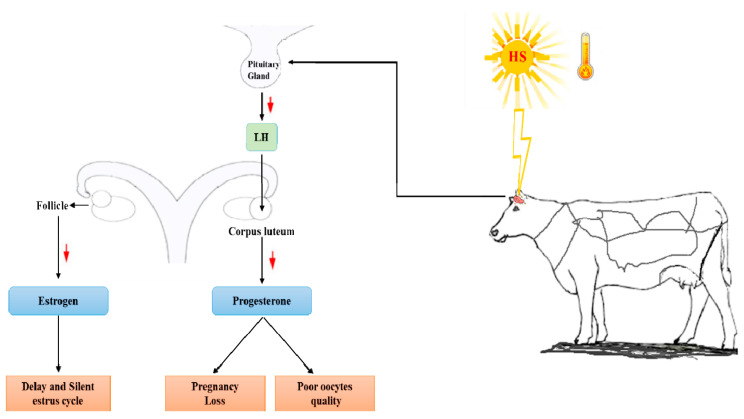
Effect of heat stress on hormonal balance and the reproduction consequences. HS adversely affects the pituitary gland, which produces an imbalance and decline in the secretion of LH, leading to reduced progesterone and estrogen levels and thus compromising reproduction through pregnancy loss and impacts on the estrous cycle, respectively. The red arrow shows the decrease of the concern hormone.

Cattle are an indispensable milk and meat production resource. Heat stress (HS) is a strong barrier to cattle reproduction and, hence, poses an alarming threat to normal life. The primary targets of HS are the pituitary gland and hormonal balance, changes in which, in turn, negatively affect the estrous cycle, follicle synthesis, and oocyte maturity and, ultimately, reduce the reproduction rates of cattle in the hot season (Figure 1 and Table 2). Although various methods and technologies, such as cooling, air conditioning, and IVF, are used to generate embryos from elite cattle, which are then stored and transferred in the favorable seasons and used to reduce the adverse effects of HS on cattle reproduction in summer, these methods are insufficient to address the deleterious effects of HS on the reproductive performance of cattle worldwide. Therefore, more focused attention should be afforded to this issue to combat the negative effects of HS on cattle reproduction.

## 13. Future Perspectives

Immediate and future priorities must be defined to mitigate the adverse effects of HS on livestock production. A wide range of available options, including proper housing, new approaches to shading, advanced cooling systems, and advanced biotechnological strategies, are required to reduce environmental stresses. The use of genome-wide association studies to identify the relationships of DNA markers linked to production and reproduction traits with HS would be a strategic approach to alleviate the effects of HS on the productivity and selection of animals with greater adaptive capability. One of the most critical research issues is to elucidate the mechanisms of HSP and the relationship between HSP tissue concentrations and livestock fertility. Additional research will be needed for the incorporation of new technologies, such as transgenesis, which could enable the genetic improvement of livestock in the future through the knock-out of undesirable genes or the knock-in of desirable genes to reduce the impact of HS on animal productivity.

## Figures and Tables

**Table 1:** Effect of heat stress (HS) on blastocyst development in vitro.

HS (°C)	Exposure Time (h)	Cleavage Rate (%)	Blastocyst Rate (%)	Reference
38.5 (control, in vitro)	22–24	86	41	[60]
40 (in vitro)	12	60	16	[57]
40.5 (in vitro)	20	75.7	18.6	[61]
41 (in vitro)	12	70	18	[59]
41 (in vitro)	12	60	13	[57]
41 (in vitro)	12	65	15	[58]
36 (in vivo, ovum pick-up)	22 May–20 July	45	6	[62]

**Table 2:** Impact of heat stress on the reproductive performance of livestock.

Female	Male
Compromised physiological function of the reproductive tract	Disturbed testicular thermoregulation mechanism
Disturbance in hormonal balance	Increased testicular temperature
Decreased estrus behavior	Damage in spermatogenesis
Decreased follicular development	Decreased semen quality
Poor-quality oocytes	Increased sperm abnormalities
Decreased fertility and conception	Decreased sperm motility
Decreased embryo development	Lower sperm concentration
Decreased embryo survival	Decreased fertility

## Data Availability

Not applicable.
